# Supplementary material for: Taxonomic diversity and functional adaptations indicated by the rhizospheric soil microbiome derived from Turkish wheat fields
Source: Biol Open. 2025 Dec 18;14(12):bio062230. doi: 10.1242/bio.062230 (PMC12755068; doi:10.1242/bio.062230)
Supplement: Supplementary information [file biolopen-14-062230-s1.pdf]

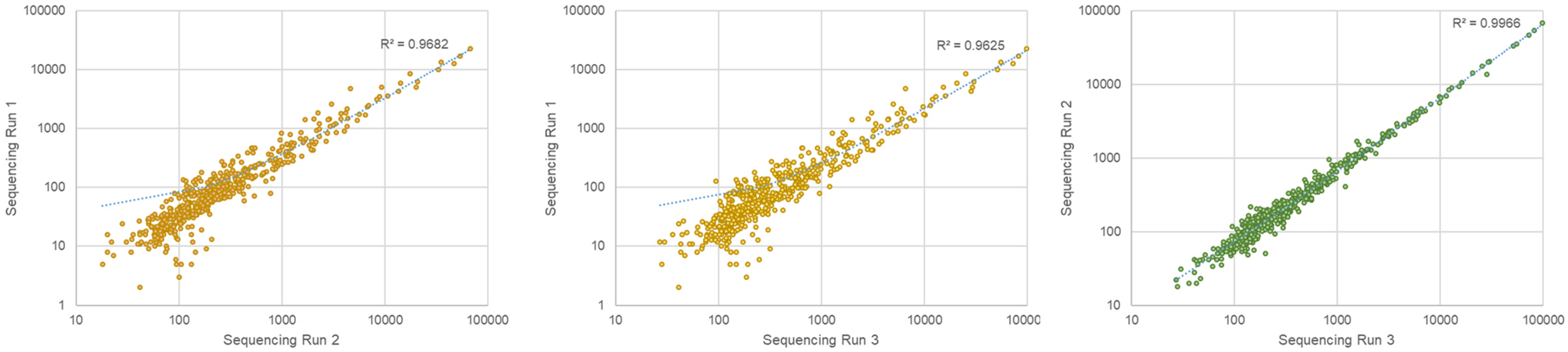

Fig. S1. Scatter plots showing pairwise comparison of abundances obtained between different sequencing runs. Each run was carried out using different barcodes for each sample and a different MinION Flow Cell; the top 500 OTUs with the highest total abundance across all samples are compared in each case, and the trendline showing linear correlation. In Run 1, a single sample (Tek2) accounted for almost 50% of all raw reads, which may have produced bias among low adundance OTUs (left & middle graphs). In Run 2 & Run 3 raw reads were more evenly distributed among all samples. The largest number of reads (1.2 million) was generated in Run 3, approximately twice as many as Run 2, but the relative abundances of the top 500 OTUs were highly consistent with each other (right).

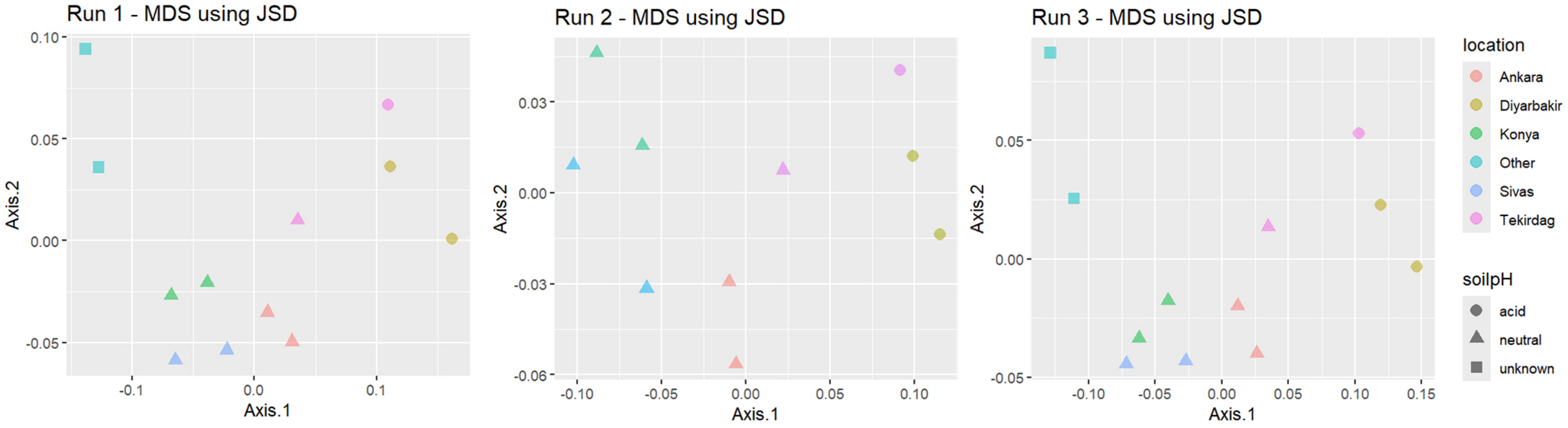

Fig. S2. Non-parametric Multi-Dimensional Scaling (NMDS) plots showing the dissimilarities between samples for 3 sequencing runs. Each run was carried out using different barcodes for each sample and a different MinION Flow Cell; run 1 & run 3 included all samples, Run 2 included all except the non-wheat soils (“Other”). Before plotting, low abundance taxa and OTUs that could not be assigned to specific taxonomic families were eliminated, as described in the Methods. Distance matrices were calculated using the Jensen Shannon Divergence (JSD) function in the vegan R package. Sample clustering was very similar in all 3 runs, with the non-wheat samples forming one distinct cluster (top left in runs 1 & 3); this cluster was significantly from the other samples by ANOSIM ($p < 0.05$). The acid soils also formed a separate cluster (on the right in all 3 plots) while in most cases pairs of samples from the same province appeared most similar to each other, but the differences between these groupings were not statistically significant.

Biology Open • Supplementary information

Table S1.

Available for download at
https://journals.biologists.com/bio/article-lookup/doi/10.1242/bio.062230#supplementary-data

Table S2.

Available for download at
https://journals.biologists.com/bio/article-lookup/doi/10.1242/bio.062230#supplementary-data

Table S3.

Available for download at
https://journals.biologists.com/bio/article-lookup/doi/10.1242/bio.062230#supplementary-data

Table S4.

Available for download at
https://journals.biologists.com/bio/article-lookup/doi/10.1242/bio.062230#supplementary-data
